# ABB Theorems: Results and Limitations in Infinite Dimensions

**DOI:** 10.1007/s10957-025-02797-z

**Published:** 2025-08-01

**Authors:** Aris Daniilidis, Carlo Alberto De Bernardi, Enrico Miglierina

**Affiliations:** 1 Institute of Statistics and Mathematical Methods in Economics, TU Wien, Wiedner Hauptstraße 8,E105-04, Wien, A-1040 Austria; 2 Dipartimento di Matematica per le Scienze Economiche, Finanziarie ed Attuariali, Università Cattolica del Sacro Cuore, Via Necchi 9, Milano, 20123 Italy

**Keywords:** ABB theorem, Efficient point, Positive functional, Density, 90C29, 46B20, 46N10

## Abstract

We construct a weakly compact convex subset of $\ell ^{2}$ with nonempty interior that has an isolated maximal element, with respect to the lattice order $\ell _{+}^{2}$. Moreover, the maximal point cannot be supported by any strictly positive functional, which shows that the Arrow-Barankin-Blackwell theorem fails. This example discloses the pertinence of the assumption that the cone has a bounded base for the validity of the result in infinite dimensions. Under this latter assumption, the equivalence of the notions of strict maximality and maximality is established.

## Introduction

Let *X* be a Banach space and *P* be a closed convex cone with base $\mathcal {B}$, that is, $\mathcal {B}$ is a closed convex subset of *P* with the property that every nonzero element $x\in P$ can be represented in a unique way in the form $x=\lambda b,$ with $\lambda >0$ and $b\in \mathcal {B}$. Notice that this is equivalent to the existence of a functional $f\in X^{*}$ that takes strictly positive values on $P\setminus \{0\}$. In what follows, we denote the set of strictly positive functionals on *P* by

$$\textrm{inn}P^{*}=\bigl \{f\in X^*:\, f(u)>0,\ \forall u\in P\setminus \{0\} \bigr \}.$$

Nonemptiness of the set $\textrm{inn}P^{*}$ clearly implies (and in finite dimensions is equivalent to) the fact that *P* is a pointed cone, that is, $P\cap (-P)=\{0\}$. Moreover, nonemptiness of the interior of $\textrm{inn}P^*$ is equivalent to the existence of a bounded base for the cone *P*.

The cone *P* defines a partial order on *X* as follows: $x\succeq y\Longleftrightarrow x-y\in P$. In case $X=\mathbb {R}^{n}$ and $P=\mathbb {R}_{+}^{n}$, the famous Arrow-Barankin-Blackwell theorem (in short, ABB theorem) asserts that every maximal element of a compact convex set can be approximated by positive elements, that is, points that support the set by means of a strictly positive functional, see [1, 18]. The result has a pertinent economic interpretation: every optimal allocation of commodities can be approximated by allocations that are supported by a nontrivial system of prices ([1, 6]). Because of its importance, a lot of effort has been devoted to extensions of the ABB result in infinite dimensional spaces. The most general result can be announced in locally convex topological vector spaces and ensures the density of the positive elements in the set of maximal elements of every compact convex set (Theorem 1). Therefore, if *X* is a Banach space equipped with a cone *P* and *K* is a compact (respectively, weakly compact) convex subset of *X*, then every maximal element of *K* can be strongly (respectively, weakly) approximated by a sequence of positive elements. Moreover, if the cone *P* has a bounded base, then the approximation is always strong, even if *K* is merely weakly compact. The same conclusion obviously holds under the assumption that the maximal element to be approximated is a point of continuity of the identity map of *K* from the weak to the norm topology (Corollary 1). However, until now, the degree of necessity of these assumptions was not sufficiently clear.

In this article, we construct an example showing that strong approximation fails in general even for the classical separable Hilbert space $\ell ^{2}$ equipped with its lattice cone $\ell _{+}^{2}$ (Proposition 4). Our counterexample concerns a weakly compact convex set with nonempty interior, framework in which the Hahn-Banach separation theorem applies, outlining that the problem stems from the fact that the lattice cone $\ell _{+}^{2}$ does not have a bounded base. Indeed, assuming that *P* has a bounded base would guarantee that a strong approximation ABB result holds for any weakly compact set. The current work shows that this assumption (or some variant of it) is essentially necessary.

Let us quote some equivalent forms of the assumption that the cone has a bounded base that have already been used in the literature, see, e.g. [13, 17, 19]: the cone *P* is of Bishop-Phelps type, the dual cone $P^{*}$ has nonempty interior, there exists a functional that strongly exposes 0 in *P* and finally that 0 is a point of continuity of the identity map of the cone *P* from the weak to the norm topology. We refer to [7] for a detailed discussion of the assumptions. We shall also show that under any of these assumptions, we can reinforce the notion of maximality: every maximal element is also strictly maximal, notion that relates to stability (see [5] and references therin).

## Notation and Preliminaries

Throughout this paper *E* stands for a locally convex topological vector space, while *X* denotes a Banach space with dual space $X^*$. If $\Im$ denotes the topology of *E*, we say that $K\subset E$ is $\Im$-compact if *K* is compact with respect to the topology $\Im$. A similar notation is used for $\Im$-convergence, $\Im$-closure, $\Im$-neighborhood, etc.

The closed unit ball, the open unit ball, and the unit sphere of *X* are denoted by $B_X$, $U_X$, and $S_X$, respectively. We also write $\delta _n\searrow 0$ to denote that $\delta _n>\delta _{n+1} >0$ ($n\in \mathbb {N}$) and $\lim _{n\rightarrow \infty }\,\delta _n=0$. Given $x\in K\subset X$, we say that $f\in X^*\setminus \{0\}$ supports *K* at *x* if $f(x)=\sup f(K)$. Finally, let us recall that if $K\subset X$ is a nonempty set, then the conical hull of *K* is the set

$$ \textrm{cone}(K)=\{x\in X: x=\lambda k,k\in K, \lambda >0\}. $$

We denote by

1 $$\begin{aligned} \textrm{Max}(K,P)=\bigl \{x\in K:\,\{x\}=K\cap (x+P)\bigr \} \end{aligned}$$

the set of *P*-maximal elements of a nonempty subset *K* of *X*, and by

2 $$\begin{aligned} \textrm{Pos}(K,P)=\bigl \{x\in K:\exists f\in \textrm{inn}\,P^{*},f(x)=\sup f(K)\bigr \} \end{aligned}$$

the set of its positive elements. Hence, $x \in \textrm{Pos}(K,P)$ if and only if there exists a functional $f\in \textrm{inn}P^*$ supporting *K* at *x*.

Let us mention that if *K* is a (weakly) compact convex set, $\textrm{Max}(K,P)$ is nonempty, as a consequence of the Zorn lemma and a standard compactness argument. Nonemptiness of $\textrm{Pos} (K,P)$ is less obvious and will follow from the forthcoming Theorem 1.

In 1953, Arrow-Barankin-Blackwell, in [1], established the density of the set $\textrm{Pos}(K,P)$ in $\textrm{Max}(K,P),$ provided *K* is a compact convex subset of $\mathbb {R}^{n}$ and $P=\mathbb {R}_{+}^{n}$. Since then this density result became relevant in Economic Theory, see [6] for updated references. General ABB results have also been obtained in arbitrary Banach spaces (see, e.g. [17, 19]) and later on in locally convex topological vector spaces ([12, 13]). The following theorem summarizes the previous results.

### Theorem 1

**(Abstract density result)** Let $(E,\Im )$ be a locally convex topological vector space, *K* a $\Im$-compact convex subset of *E*, and *P* a closed convex cone with base $\mathcal {B}$. Then

3 $$\begin{aligned} \textrm{Pos}(K,P)\subseteq \textrm{Max}(K,P)\subseteq \overline{\textrm{Pos} (K,P)}^{\Im }. \end{aligned}$$

For the convenience of the reader we provide a detailed proof of the previous theorem, based on the notion of dilating cones (see [4]), in the special case in which $E=X$ is a Banach space and $\Im$ is either the weak or the norm topology on *X*, which is actually the case relevant in this work. The same arguments can be easily adapted to locally convex topological vector spaces, and for a detailed proof of Theorem 1 in this more general setting, we refer to [12, Theorem 3].

### Proof

Let us denote by $\Im$ the norm or the weak topology on a Banach space *X*. Assume that *K* is a $\Im$-compact convex subset of *X*, $P=\overline{\textrm{cone}}^{\Im }(\mathcal {B})$ and $\bar{x}\in \textrm{Max}(K,P)$, that is, $\{\bar{x}\}=K\cap (\bar{x}+P)$. By the Hahn-Banach theorem, there exists $f\in S_{X^*}$ such that $\alpha :=\inf f(\mathcal {B})>0$. Notice that if $\delta \in (0,\alpha )$ then $\inf f(\mathcal {B}+\delta B_X)=\alpha -\delta >0$. It easily follows that the cone $\widetilde{P}=\overline{\textrm{cone}}^{\Im }(\mathcal {B}+\delta B_X)$ admits, for any positive $\theta$, the $\Im$-closed convex set $\{x\in \widetilde{P}:\, f(x)=\theta \}$ as a base. Let $\{\delta _n\}\subset (0,\alpha )$ be such that $\delta _n\searrow 0$ and consider the closed convex cones

$$\begin{aligned} P_{n}=\overline{\textrm{cone}}^{\Im }(\mathcal {B}+\delta _n B_X),\qquad \qquad (n\in \mathbb {N}). \end{aligned}$$

By our choice of $\{\delta _n\}$, $P_{n}$ has a base whenever $n\in \mathbb {N}$. Moreover, we claim that $P=\bigcap _{n\ge 1}P_{n}$. To prove the claim, take $z\in \bigcap _{n\ge 1}P_{n}$ and let prove that $z\in P$. Since $z\in \bigcap _{n\ge 1}P_{n}$, there exist sequences $\{\lambda _n\}\subset [0,\infty )$, $\{b_n\}\subset B_X$, and $\{w_n\}\subset \mathcal {B}$ such that $\Vert z- \lambda _n w_n-\lambda _n \delta _n b_n\Vert \rightarrow 0$. If $f\in S_{X^*}$ is defined as above, we have

$$f(z)=\lim _n\lambda _n\bigl (f(w_n)+\delta _nf(b_n)\bigr )\ge \limsup _n \lambda _n(\alpha -\delta ),$$

and hence the sequence $\{\lambda _n\}$ is necessarily bounded. Since $\Vert \lambda _n \delta _n b_n\Vert \rightarrow 0$, we have $z\in \overline{P}^\Im =P$, and our claim is proved.

For each $n\ge 1$, by a plain application of Zorn’s Lemma, we can find a $P_{n}$-maximal point $x_{n} \in \textrm{Max}(K,P_{n})$, such that

$$\begin{aligned} x_{n}\in K_{n}:=K\,\bigcap \,(\bar{x}+P_{n}). \end{aligned}$$

Notice that $P=\bigcap _{n\ge 1}P_{n}$ readily yields $\{\bar{x}\}=\bigcap _{n\ge 1}K_{n}$. Since $\{K_n\}$ is a decreasing sequence of $\Im$-compact subsets of *X* such that $\{\bar{x}\}=\bigcap _{n\ge 1}K_{n}$, for every $\Im$-neighborhood *V* of $\bar{x}$ we have that there exists $n_0\in \mathbb {N}$ such that $K_{n_0}\subset V$ (see, e.g., [8, Fact 1.6]). Hence,

$$\begin{aligned} \Im \text {-}\underset{n\rightarrow \infty }{\lim }x_{n}=\bar{x}. \end{aligned}$$

Since $\{x_{n}\}=K\cap (x_{n}+P_{n})$ and $P_{n}$ has nonempty interior in the norm topology, there exists a functional $x^{*}\in P_{n}^{*}$ that supports the set *K* at the point $x_{n}$. Since $x^{*}$ is actually a strictly positive functional for the original cone *P*, the proof is complete.$\square$

### Remark 1

Let us mention that in [15] the authors, working with a refinement of the notion of *P*-maximality (Henig proper maximality), were able to obtain a result, namely [15, Theorem 4.1], in the spirit of Theorem 1 but under weaker assumptions: nonconvex sets *K* have been considered and the assumption of $\Im$-compactness was replaced by $\Im$-asymptotic compactness (see [15, Definition 2.1] for the definition of $\Im$-asymptotic compactness).

In a Banach space *X*, Theorem 1 simultaneously expresses two different density results, for the norm and, respectively, for the weak topology. Notwithstanding, assuming norm compactness is very restrictive in infinite dimensions, on the other hand, concluding that only weak approximation holds is suboptimal. Therefore, it is desirable to obtain a strong approximation result for weakly compact sets. However, this was achieved only under additional assumptions. Jahn [17] was the first to derive a norm approximation result for weakly compact subsets, assuming that the cone *P* was of “Bishop-Phelps type”. Subsequently, Petschke [19] (see also [13] for a different approach) refined Jahn’s proof to conclude the same result for cones *P* having a bounded base. More recent related references include [14–16].

We resume these results in a general scheme presented below. To this end, let us recall the following definition.

### Definition 1

**(Point of continuity)** Let *A* be a nonempty subset of a Banach space *X*. We say that $\bar{x}\in A$ is a *point of continuity* for the set *A* and we denote $\bar{x}\in \textrm{PC}\bigl (A,(w,\Vert \cdot \Vert )\bigr )$, if the identity mapping

$$\textrm{Id}:(A,w)\rightarrow (A,\Vert \cdot \Vert )$$

is continuous at $\bar{x}$.

It is well-known (see, e.g. [7]) that a closed convex pointed cone *P* has a bounded base if and only if $0\in \textrm{PC} \bigl (P,(w,\Vert \cdot \Vert )\bigr ).$ In view of the above, a careful investigation of the proof of Theorem 1 leads readily to the following corollary (see also [3, Theorem 3.1]).

### Corollary 1

**(Density result with combined topologies)** Let *K* be a nonempty *w*-compact convex subset of *X*, *P* a convex closed cone with base and let $\overline{x} \in \textrm{Max}(K,P)$. Then $\overline{x}\in \overline{\textrm{Pos} (K,P)}^{\parallel \cdot \Vert }$ provided that one of the following conditions is satisfied:

(i) $0\in \textrm{PC}\bigl (P,(w,\Vert \cdot \Vert )\bigr )$ (equivalently, *P* has a bounded base);
(ii) $$\overline{x}\in \textrm{PC}\bigl (K,(w,\Vert \cdot \Vert )\bigr )$$.

## Main Results

The main results are twofold. In the first subsection, we consider the same framework as in Corollary 1 (where the strong density result holds) and show that in this case, every $\bar{x}\in \textrm{Max}(K,P)$ is also a strictly maximal element, see forthcoming definition in (4). The latter is a more restrictive notion of maximality introduced to study stability and well-posedness in vector optimization (see [2, 5] and references therein).

In the second subsection, we show that the ABB density result fails for general weakly compact convex sets in a separable Hilbert space where the ordering cone *P* is the natural lattice cone. The reason is the lack of a bounded base for this cone.

### Relation Between $\textrm{Max}(K,P)$ and $\textrm{StMax}(K,P)$

Let us start with the definition of strict maximality ([2, 5]):

4 $$\begin{aligned} \textrm{StMax}(K,P)=\bigl \{x\in K:\,\forall \varepsilon>0,\exists \delta >0,(P+\delta B_{X})\cap (K-x)\subset \varepsilon B_{X} \bigr \}. \end{aligned}$$

**Fig. 1:**
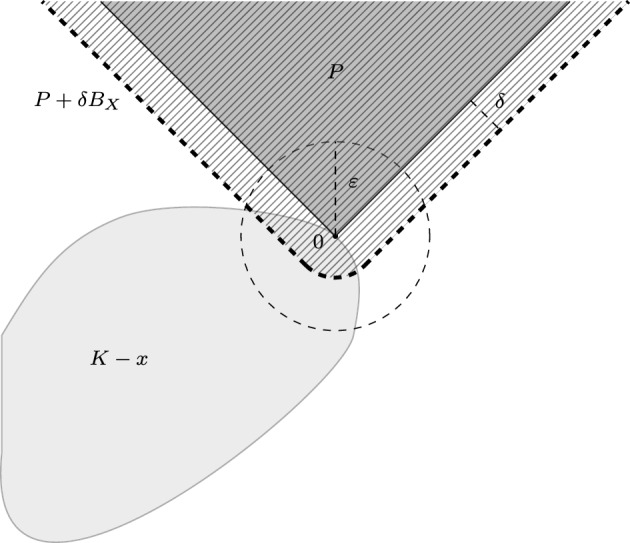
Definition of strict maximality: $x \in \textrm{StMax}(K,P)$

The above definition is illustrated by Figure 1. It is easy to see that every strictly maximal point is maximal, but the converse is not true (see forthcoming Proposition 1 for example). However, under the assumption of Corollary 1, we will show that $\textrm{Max}(K,P)$ and $\textrm{StMax}(K,P)$ coincide and they are both nonempty. To do so, we need to recall the following result stated in a general locally convex space, see [2, Theorem 2.2.1]

#### Theorem 2

**(Strict maximality in locally convex spaces)** Let *E* be a locally convex topological vector space, $K\subset E$ compact convex, and *P* a closed convex cone with base. Suppose that $x\in \textrm{Max}(K,P)$. Then for each neighbourhood $\mathcal {W}$ of the origin there exist a neighbourhood $\mathcal {V}$ of the origin such that

$$(P+\mathcal {V})\cap (K-x)\subset \mathcal {W}.$$

Notice that for the special case in which $E=X$ is a Banach space considered with its norm topology, we deduce from Theorem 2 that $\textrm{Max}(K,P)$ coincides with $\textrm{StMax}(K,P)$, whenever *K* is (norm) compact. This is of course a very restrictive assumption in infinite dimensions. The following result remedies partially this inconvenience.

#### Theorem 3

Let *K* be a *w*-compact convex subset of *X*, *P* a closed convex cone with base, and $\overline{x}\in \textrm{Max}(K,P)$. Then $\overline{x}\in \textrm{StMax}(K,P)$ provided one of the following conditions is satisfied:

(i) $0\in \textrm{PC}\bigl (P,(w,\Vert \cdot \Vert )\bigr )$ (equivalently, *P* has a bounded base);
(ii) $$\overline{x}\in \textrm{PC}\bigl (K,(w,\Vert \cdot \Vert )\bigr )$$.

#### Proof

We may suppose without any loss of generality that $\overline{x}=0$. We first prove the conclusion in the case in which (i) holds. Let $\varepsilon >0$ and take a base $\mathcal {B}$ of *P* contained in $\frac{\varepsilon }{3} B_X$. By the Hahn-Banach theorem, there exist a functional $f\in S_{X^*}$ and a real number $\alpha$ such that

$$0=f(0)\le \sup f(K)<\alpha \le \inf f(\mathcal {B})\qquad \text {(see Figure~2).}$$

**Fig. 2:**
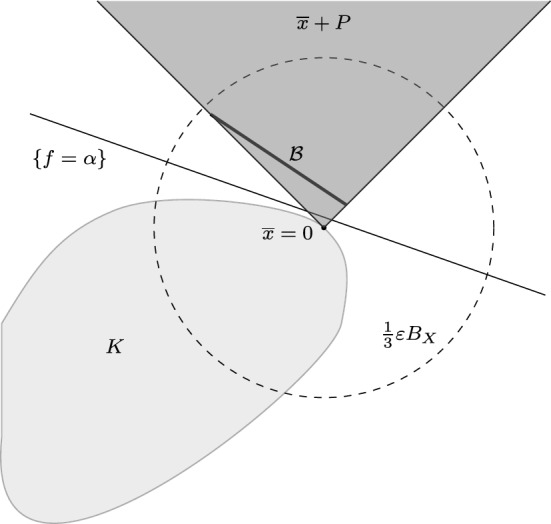
Proof of Theorem 3(i)

Let $0<\delta \le \min \{\,\alpha ,\, \varepsilon /3\,\}$ and let us show that $(P+\delta B_X)\cap K\subset \varepsilon B_X$. To do this, let $y=p+\delta b\in K$ with $p\in P$ and $b\in B_X$ and observe that

$$f(p)=f(y-\delta b) <\alpha +\delta \le 2\alpha .$$

It follows that $f(p/2)\le \alpha$, therefore there exists a real number $\lambda \in [0,1]$ such that $\frac{p}{2}\in \lambda \mathcal {B}$. By recalling that $\mathcal {B}\subset \frac{\varepsilon }{3}B_X$, we have that $\frac{p}{2}\in \frac{\varepsilon }{3}B_X$ and consequently

$$\Vert y\Vert \le \Vert p\Vert +\delta \le \varepsilon .$$

The conclusion follows.

Let us now suppose that (ii) holds and let us consider $\varepsilon >0$. Since

$$0\in \textrm{PC}\bigl (K,(w,\Vert \cdot \Vert )\bigr ),$$

there exists a *w*-neighbourhood $\mathcal {W}$ of the origin such that

$$0\in \mathcal {W}\cap K\subset \varepsilon B_X.$$

By Theorem 2, there exists a *w*-neighbourhood (in particular, a norm neighbourhood) of the origin $\mathcal {V}$ such that

$$ (P+\mathcal {V})\cap K\subset \mathcal {W}\cap K\subset \varepsilon B_{X}, $$

and the proof is complete.$\square$

The following proposition shows that neither of the assumptions (i), (ii) in Theorem 3 can be removed.

#### Proposition 1

Let $$X=\ell ^2$$. Define

$$ P=\{x=(x_n)\in X:\, nx_1-|x_n|\ge 0,\ \forall n\ge 2\}\quad \text {and}\quad K=-P\cap B_X.$$

Then

$$\textrm{Max}(K,P)=\textrm{Pos}(K,P)=\{0\}\quad \text {and}\quad \textrm{StMax}(K,P)=\emptyset .$$

#### Proof

Since the cone is pointed, we have $P\cap K\subset P\cap (-P)=\{0\}$ and consequently $0\in \textrm{Max}(K,P)$. We claim that the origin is the unique element of the set $\textrm{Max}(K,P)$. Indeed, if $\overline{x}\in K\setminus \{0\}$ then $\overline{x}\in -P$ and consequently $0\in \overline{x}+P$, proving the claim. Moreover, it is easily seen that $e_1\in \textrm{inn}\,P^*$ and $\sup e_1(K)=0$. Therefore we deduce that $0\in \textrm{Pos}(K,P)$.

Let us now prove that $0\not \in \textrm{StMax}(K,P)$. To do this, let $n\in \mathbb {N}$ and define the sequence

$$z^n=(z^n_k)_{k}\in \ell ^2$$

by

$$\textstyle z^n_k={\left\{ \begin{array}{ll} -\frac{1}{2 (n+1)}\,, & \text {if}\ k=1, \\ \phantom {--}{\frac{1}{2}}\,, & \text {if}\ k= n+1, \\ \phantom {--}0\,, & \text {if}\ k\notin \{1, n+1\}. \end{array}\right. }$$

Then it is easy to see that $\{z^n\}_n\subset K$. For every $n\ge 1$, let us set

$$\begin{aligned} w^n_1=-z^n_1=\frac{1}{2(n+1)} \quad \text {and}\quad w^n_k=z^n_k,\,\text { for }\, k\ne 1. \end{aligned}$$

Then $\{w^n\}_n \subset P$ and $d(z^n, P)\le \Vert z^n-w^n\Vert =\frac{1}{n+1} \rightarrow 0$ (as $n\rightarrow \infty$) whereas

$$\Vert z^n\Vert ^2=\frac{1}{4(n+1)^2}+\frac{1}{4}\ge \frac{1}{4},\quad \text {for all }\, n\ge 1.$$

The claim follows.$\square$

We shall now present an example showing that in Theorem 3, weak compactness of the set *K* cannot be replaced by the weaker assumption that the set *K* is weakly closed and bounded. It is a slight modification of [5, Example 4.6].

#### Example 1

Let $X=\ell ^1$ equipped with its lattice cone $P=\ell ^1_+$. It is worth pointing out that *P* has both bounded and unbounded bases. Indeed, let us consider a strictly positive functional $f=(f_n)_{n\ge 1}\in \ell ^\infty _{++}\equiv \textrm{inn}(\ell ^{1}_{+})^{*}$. If there exists $\alpha >0$ such that $f_n\ge \alpha$ for every $n\ge 1$, then $f^{-1}(1)\cap P$ is a bounded base for $\ell ^1_+$, otherwise *f* generates an unbounded base. In particular, taking a functional of the latter type:

$$\begin{aligned} f:=(1/n)_{n\ge 1} \in \textrm{inn}(\ell ^1_+)^{*} \end{aligned}$$

the set $f^{-1}(1)\cap P$ is an unbounded base for *P*. We fix this cone and consider the closed convex bounded set

$$\begin{aligned} K=\{x\in \ell ^1: -1\le f(x) \le 0\} \,\bigcap \, 2B_X. \end{aligned}$$

It follows directly that $0\in \textrm{Pos}(K,P)$ and *a fortiori* $0\in \textrm{Max}(K,P)$.

On the other hand, taking $x^n:= e_n - \frac{1}{n} e_1\in \frac{1}{n}B_X+P$, for all $n\in \mathbb {N}$, where $\{e_n \}_n$ is the standard unit-vector basis of $\ell ^1$, we deduce that $\{x^n\}_n \subset K$ and $\Vert x^n \Vert \ge 1$ for every $n\in \mathbb {N}$. This shows that $0\notin \textrm{StMax}(K,P)$.

The following proposition shows that if the cone *P* has a bounded base (as was the case in the previous example), the set $\textrm{StMax}(K,P)$ is always nonempty. Therefore, in Example 1, the set of strict maxima is nonempty (and strictly contained in $\textrm{Max}(K,P)$). This being said, in forthcoming Proposition 3 we shall see that if *P* does not have a bounded base, the set of strict maxima can be empty. The key idea of our construction is to define *K* as a suitable equivalent unit ball of the Banach space $c_0$, to do this we use some well-known renorming techniques (see, e.g.,[9, 10] and the references therein for some related results in this field).

We recall that a functional $f\in X^*\setminus \{0\}$ is called a supporting functional of *K* at $x_0$ if $f(x_0)=\sup f(K)$.

#### Proposition 2

**(existence of strict maxima for a bounded based cone)** Let *K* be a nonempty closed convex bounded subset of a Banach space *X* and *P* a convex closed cone with a bounded base. Then $\textrm{StMax}(K,P)\ne \emptyset$.

#### Proof

Since *P* has a bounded base, then $\textrm{int}P^{*}$ is nonempty. Hence, by Bishop-Phelps theorem (see, e.g., [11, Theorem 7.41]), there exists $x^*_0\in \textrm{int}P^{*}$ that is a supporting functional for *K* at a point $x_0\in K$, i.e., $x_0 \in \textrm{Pos}(K,P)$. By [5, Corollary 5.4], we conclude that $x_0\in \textrm{StMax}(K,P)$.$\square$

Let us further denote by $c_0:=\{x=(x_n)_n\in \mathbb {R}^{\mathbb {N}}:\, \underset{n\rightarrow \infty }{\lim }\ x_n =0 \}$ the Banach space of all null real valued sequences equipped with the norm $\Vert x\Vert _{\infty }:=\underset{n\ge 1}{\sup }\,|x_n|$. Let us consider the linear operator $T:c_0\rightarrow \ell ^2$ defined for every $x\in c_0$ as follows:

5 $$\begin{aligned} x:=(x_n) \,\mapsto \, T(x):= \left( \frac{x_n}{2^n}\right) _{n\ge 1}\in \ell ^{2}. \end{aligned}$$

Notice that *T* is injective, continuous and

$$\begin{aligned} \Vert Tx\Vert _2= \sqrt{\sum _{n\ge 1}\frac{|x_n|^2}{4^n}} \, \le \, \left( 1/\sqrt{3}\right) \, \Vert x\Vert _{\infty }\,. \end{aligned}$$

Therefore,

6 $$\begin{aligned} |||x||| \,:=\,\Vert x\Vert _{\infty } \, + \, \Vert Tx\Vert _{2} \end{aligned}$$

is an equivalent norm on $c_0$.

We are now ready to provide an example in which the set $\textrm{StMax}(K,P)$ is empty, even if $\textrm{Max}(K,P)$ is nonempty, showing the pertinence of the assumption that the cone *P* has a bounded base in Proposition 2.

#### Proposition 3

**(example where** $\textrm{StMax}(K,P)$ **is empty)** Consider the Banach space $X=(c_0,|||\cdot |||)$, where $|||\cdot |||$ is the equivalent norm defined in (6). Consider further the closed convex bounded set $K=B_X$ and the lattice cone

$$P:=(c_0)_+ =\{ x=(x_n)\in c_0:\, x_n\ge 0,\ \text {for all } n\ge 1 \}.$$

Then

$$\begin{aligned} \textrm{StMax}(K,P)=\emptyset \qquad \text {and} \qquad \textrm{Max}(K,P)\ne \emptyset . \end{aligned}$$

#### Proof

Let us denote by $\{e_n\}_n$ the canonical basis of $c_0$ and set $\alpha := 1+1/\sqrt{3}$. It follows that

$$\Vert x\Vert _{\infty }\, \le |||x||| \, \le \alpha \,\Vert x\Vert _{\infty },\quad \text {for all } x\in c_0. $$

Set $\bar{x}:=\frac{2}{3} e_1$ and notice that for all $p\in P\setminus \{0\}$ we have $|||\bar{x}||| = 1 < |||\bar{x} + p |||$. It follows readily that $\bar{x} = \frac{2}{3} e_1\in \textrm{Max}(K,P)$, therefore the set of maxima of *K* is not empty.

It remains to show that $\textrm{StMax}(K,P)=\emptyset$. It is sufficient to prove that no point in the boundary $S_X$ of *K* can be in $\textrm{StMax}(K,P)$.

To this end, let $x=(x_n)\in S_X$ (that is, $|||x|||=1$). Since $\textrm{StMax}(K,P)\subset \textrm{Max}(K,P)$ we can clearly also assume that $x\in \textrm{Max}(K,P)$. Therefore

$$\begin{aligned} |||x|||=1 \, < \, |||x+ p|||,\quad \text { for all }\, p\in P\setminus \{0\}. \end{aligned}$$

Notice further that $\Vert x\Vert _{\infty }\ge 1/{\alpha }$. Therefore, since $x\in c_0$, there exists $n_0\in \mathbb {N}$ such that $|x_n|\le 1/{2\alpha }$, for all $n\ge n_0$. Setting

$$p_n:=(1/2{\alpha })\,e_n \in P$$

we have:

$$\begin{aligned} \textstyle |||x|||\,=\,1 \,< \, |||x+p_n||| =\, \Vert x +\frac{1}{2\alpha }e_n\Vert _{\infty } \, +\,\left( \underset{k\in \mathbb {N}\setminus \{n\}}{\sum } (x_k^2/4^k) \,+\,\frac{1}{4^n}\left( \,x_n+\frac{1}{2\alpha }\right) ^2\right) ^{1/2}. \end{aligned}$$

Notice that $\Vert x +\frac{1}{2\alpha }e_n\Vert _{\infty }=\Vert x\Vert _{\infty }$, for $n\ge n_0$ and that $\beta _n:=|||x+\frac{1}{2\alpha }e_n|||\rightarrow 1$. Set

$$z_n=\frac{1}{\beta _n}(x+p_n)\in B_X\equiv K$$

and notice that $d(z_n,x+P)\,\le \, |||z_n-(x+p_n)|||\rightarrow 0$ (since $\beta _n\rightarrow 1$).

On the other hand, by the triangular inequality we obtain

$$\begin{aligned} |||(x+p_n)-z_n|||\, +\, |||z_n-x |||\, \ge \, |||p_n||| \, >\, \Vert p_n\Vert _{\infty } = \frac{1}{2\alpha }\,. \end{aligned}$$

Since $|||(x+p_n)-z_n||| \rightarrow 0$, we deduce that $z_n-x \notin (1/2\alpha )U_X$, for all *n* sufficiently large, whence $x\not \in \textrm{StMax}(K,P)$.$\square$

### Failure of Approximation of $\textrm{Max}(K,P)$ by $\textrm{Pos}(K,P)$

In this subsection, we construct an example showing that the strong version of the ABB theorem fails to hold. This outlines the pertinence of the assumptions of all known infinite dimensional versions of the result, revealing in particular that the assumptions in Corollary 1 cannot be removed.

Indeed, we construct a weakly compact convex set in $\ell ^2$ with an isolated maximal point that is not a point of continuity and cannot be supported by a strictly positive functional with respect to the natural ordering cone $\ell ^2_+$. It is worth pointing out that the set *K* in this example will have nonempty interior. On the other hand, the cone *P* does not (and cannot) have any bounded base.

#### Proposition 4

**(failure of ABB strong approximation)** Let $X=\ell ^{2}$, $P=\ell _{+}^{2}$, and

$$ K=\{x=(x_n)\in X:\, x_1+x_n^2\le 0,\ \forall n\ge 2\}\cap B_X.$$

Then

$$\textrm{Max}(K,P)\cap U_X=\{0\}\quad \text {and}\quad \textrm{StMax}(K,P)\cap U_X=\textrm{Pos}(K,P)\cap U_X=\emptyset .$$

#### Proof

First of all, let us note that if $x=(x_n)\in K\setminus \{0\}$, then by the definition of *K*, we have $x_1<0$. On the other hand, we readily have $0\in \textrm{Max}(K,P)$.

Now, let us prove that $\textrm{Max}(K,P)\cap U_X=\{0\}$. To do this, let $x=(x_n)\in K \setminus \{0\}\cap U_X$. Then the set

$$\mathcal {N}_x:=\{n\in \mathbb {N}:\, n\ge 2,\, x_n^2=|x_1| \}$$

is finite (and possibly empty). Take $n_0>1$ such that $n_0\not \in \mathcal {N}_x$. Then for $\varepsilon >0$ sufficiently small we have $|x_1|>(|x_{n_0}|+\varepsilon )^2$ and $x_{n_{0}} \pm \varepsilon \in U_{X}$. It follows that the segment

$$J:=[x-\varepsilon e_{n_0},x+\varepsilon e_{n_0}]\subset K,$$

and consequently $x\not \in \textrm{Max}(K,P)$, since necessarily $x+P$ intersects $J\setminus \{x\}$.

We claim that $0\not \in \textrm{Pos}(K,P)$. Indeed, let $y=(y_n)\in \textrm{inn}\,P^*=\ell ^2_{++}$ be arbitrarily chosen. Then taking $\alpha >0$ sufficiently small, the point $x=(-\alpha , \sqrt{\alpha },0,\ldots )$ belongs to *K* and $\sup y(K)\ge y(x)=-\alpha y_1+\sqrt{\alpha }y_2>0=y(0)$, showing that *y* cannot support *K* at 0.

Let us now claim that $0\not \in \textrm{StMax}(K,P)$. To prove this, let $n\in \mathbb {N}$ and define $z^n=(z^n_k)_k\in \ell ^2$ by

$$\textstyle z^n_k={\left\{ \begin{array}{ll} -\frac{1}{\sqrt{2}}\,\frac{1}{n}, & \text {if}\ k=1, \\ \phantom {-}\frac{1}{\sqrt{2}}\,\frac{1}{\sqrt{n}}, & \text {if}\ k\in \{2,\ldots , n+1\}, \\ \phantom {--}0, & \text {if}\ k>n+1. \end{array}\right. }$$

Observe that

$$\begin{aligned} 1\ge \Vert z^n\Vert ^2=\frac{1}{2n^2}+n\frac{1}{2n}\ge \frac{1}{2}\qquad \qquad (n\in \mathbb {N}), \end{aligned}$$

and that, by definition of *K*, we clearly have $\{z^n\}\subset K$. Moreover, setting $w^n_k=\max \{z^n_k,0\}$ for all $n,k\ge 1$, we have $\{w^n\}_n\subset P$ and $d(z^n, P)\le \Vert z^n-w^n\Vert \rightarrow 0$ (as $n\rightarrow \infty$). Therefore the claim follows.

The fact that

$$\textrm{StMax}(K,P)\cap U_X=\textrm{Pos}(K,P)\cap U_X=\emptyset $$

follows directly from the inclusion

$$\textrm{StMax}(K,P)\cup \textrm{Pos}(K,P)\subset \textrm{Max}(K,P).$$

The proof is complete. $\square$

#### Remark 2

It is well-known that the closed unit ball *B* of $\ell ^2$ has the Kadets-Klee property, which guarantees that every boundary point is a point of continuity from the weak to the norm topology. The set *K* in Proposition 4 is a subset of *B*, containing the basic vector $e_1:=(1,0,\dots )$ and constructed in a way that the part around $e_1$ is sufficiently flattened so that the Kadets-Klee property fails and at the same time there is no other maximal element near $e_1$.

## Data Availability

Our manuscript has no associated data.
